# Intraoperative sensor technology quantifies inter-prosthesis pressure for predicting lower limb alignment after Oxford unicompartmental knee arthroplasty

**DOI:** 10.3389/fbioe.2023.1210713

**Published:** 2023-08-09

**Authors:** Juncheng Ge, Xiaowei Sun, Changquan Liu, Qidong Zhang, Bailiang Wang, Wanshou Guo

**Affiliations:** ^1^ Department of Orthopaedic Surgery, Peking University China-Japan Friendship School of Clinical Medicine, Beijing, China; ^2^ Department of Orthopaedic Surgery, China-Japan Friendship Hospital, Beijing, China; ^3^ China-Japan Friendship Hospital, Institute of Clinical Medical Sciences, Chinese Academy of Medical Sciences and Peking Union Medical College, Beijing, China

**Keywords:** Oxford unicompartmental knee arthroplasty (UKA), limb alignment, posterior tibial slope (PTS), pressure sensor, hip-knee-ankle angle (HKAA)

## Abstract

**Purpose:** The aim of this study is to quantify inter-prosthetic pressures at different knee angles in Oxford unicompartmental knee arthroplasty (OUKA) and its correlation with postoperative lower limb alignment.

**Methods:** This study included 101 patients (122 knees) who underwent OUKA from March 2022 to July 2022. The previously designed matrix flexible force sensor was used to measure the inter-prosthesis pressure of different knee joint angles during the UKA operation, and the force variation trend and gap balance difference were obtained. The correlation between inter-prosthesis pressure and postoperative lower limb alignment index including hip-knee-ankle angle (HKAA) and posterior tibial slope (PTS) was analyzed. The effect of PTS change (ΔPTS) on the inter-prosthesis pressure and the range of motion (ROM) of the knee joint was analyzed. Radiographic and short-term clinical outcomes of included patients were assessed.

**Results:** The inter-prosthesis pressure of the different knee joint angles during the operation was not consistent. The mean inter-prosthesis pressure and gap balance difference were 73.68.28 ± 41.65N and 36.48 ± 20.58N. The inter-prosthesis pressure at 0° and 20° was positively correlated with postoperative HKAA (*p* < 0.001). ΔPTS was positively correlated with the pressure at the end of knee extension and negatively correlated with the pressure at the end of knee flexion (*p* < 0.001). The HKAA, ROM, degree of fixed knee flexion deformity, and knee society score of the included patients were significantly improved compared with those before the operation (*p* < 0.001).

**Conclusion:** The inter-prosthesis pressure measured at the knee extension position can predict postoperative HKAA to some degree. Changes in PTS will affect the inter-prosthesis pressure at the end of flexion and end of knee extension, but this change is not related to the range of motion of the knee joint.

## 1 Introduction

Oxford unicompartmental knee arthroplasty (OUKA) is an effective treatment for end-stage knee osteoarthritis, especially for anteromedial osteoarthritis, with the advantages of less trauma and faster recovery (Mohammad et al., 2018; Hansen et al., 2019; Walker et al., 2019). OUKA repairs the diseased knee joint through osteotomy and prosthesis filling, and restores the tension of the soft tissue around the knee joint (especially the medial collateral ligament). The restoration of soft tissue tension is closely related to the effect of surgery and the dynamic stability of the knee joint. However, whether it is total knee arthroplasty (TKA) or UKA, the intraoperative judgment of soft tissue tension mostly depends on the operator’s experience, and it is difficult to be directly quantified. In some respects, the use of sensors to measure inter-prosthesis pressure can indirectly reflect soft tissue tension. Sensor technology has been reported to balance the medial-lateral space and the flexion-extension space in TKA, allowing for more precise surgery (Chow et al., 2018; van der Linde et al., 2018). The potential benefit of sensors is the ability to obtain real *in vivo* measurements, and smart implants embedded with sensor technology offer the opportunity to improve surgical outcomes (Kelmers et al., 2023). For OUKA, the soft tissue tension and the balance of flexion and extension are particularly important (Heyse et al., 2016; Kwon et al., 2020). Unfortunately, there are few reports of sensor technology used in OUKA [(Mentink et al., 2017; Becker et al., 2013)], and the standard of flexion-extension gap balance has not been established.

Lower limb alignment is an important indicator, it can reflect whether the mechanical support structure of the knee joint has recovered after UKA. Postoperative coronal lower extremity alignment (i.e., hip-knee-ankle angle, HKAA) is directly related to surgical outcome, particularly long-term outcome. Intraoperative overfilling of the medial compartment can lead to valgus and accelerate degeneration of the lateral compartment, which is the commonest reason for UKA failure (Slaven et al., 2020; Slaven et al., 2021; Kamenaga et al., 2022). The optimal coronal alignment after medial UKA is considered to be neutral or mildly varus (Inoue et al., 2016; Plancher et al., 2022), and the causes of valgus may be related to the following: inaccurate determination of inter-prosthetic pressures during surgery; or surgeons implanting an oversized spacer for fear of dislocation (Ro et al., 2018; Misir et al., 2020). On the sagittal plane, the posterior tibial slope (PTS) has a significant impact on the surgical effect and prosthesis life (Hernigou and Deschamps, 2004; Gulati et al., 2009; Suero et al., 2012; Lee et al., 2014). A large number of studies have shown that changes in PTS can significantly affect the flexion-extension gap and postoperative range of motion in cruciate-retaining total knee arthroplasty. However, there are still very limited studies on the impact of PTS changes on inter-prosthesis pressure during UKA surgery and postoperative motor function (Takayama et al., 2016; Koh et al., 2021; Khow et al., 2022).

To help the operator to evaluate the soft tissue tension more accurately during the operation, we used a pressure sensor to measure the inter-prosthesis pressure of different knee angles during the OUKA operation. At the same time, we further analyzed its correlation with postoperative lower limb alignment.

## 2 Materials and methods

### 2.1 General information

This study prospectively enrolled 101 patients (122 knees) undergoing OUKA from March 2022 to July 2022. The prosthesis used in all patients was the phase III Oxford mobile-bearing prosthesis, hybrid type (Biomet, Bridgend, United Kingdom). The basic information about the included patients is shown in Table 1.

**TABLE 1:** Patient demographics.

Demographic	
No. of cases	101 (122 knees)
Gender (Male/female)	17/84
Age	66.75 ± 5.96
Side (L/R)	54/68
BMI(body mass index)	27.63 ± 3.74
Size of femoral component (XS/S/M/L)	4/92/22/4
Size of tibial component (AA/A/B/C/D/E)	9/41/45/23/2/2
Size of polyethylene bearing (3/4/5/6)	56/43/17/15

Inclusion criteria were radiographically diagnosed isolated medial compartment osteoarthritis or idiopathic osteonecrosis, fixed flexion deformity <15°, knee range of motion (ROM) greater than 90°, and varus deformity <15°. Patients with any of the following criteria were excluded from the study: 1) knee osteoarthritis involving the lateral compartment; 2) inflammatory arthritis; 3) incomplete clinical and radiological records. The study was approved by the ethics committee (approval number:2020-50-K28) and was in accordance with the Declaration of Helsinki. All the patients included in the studies have signed the informed consent.

### 2.2 Sensor

**FIGURE 1:**
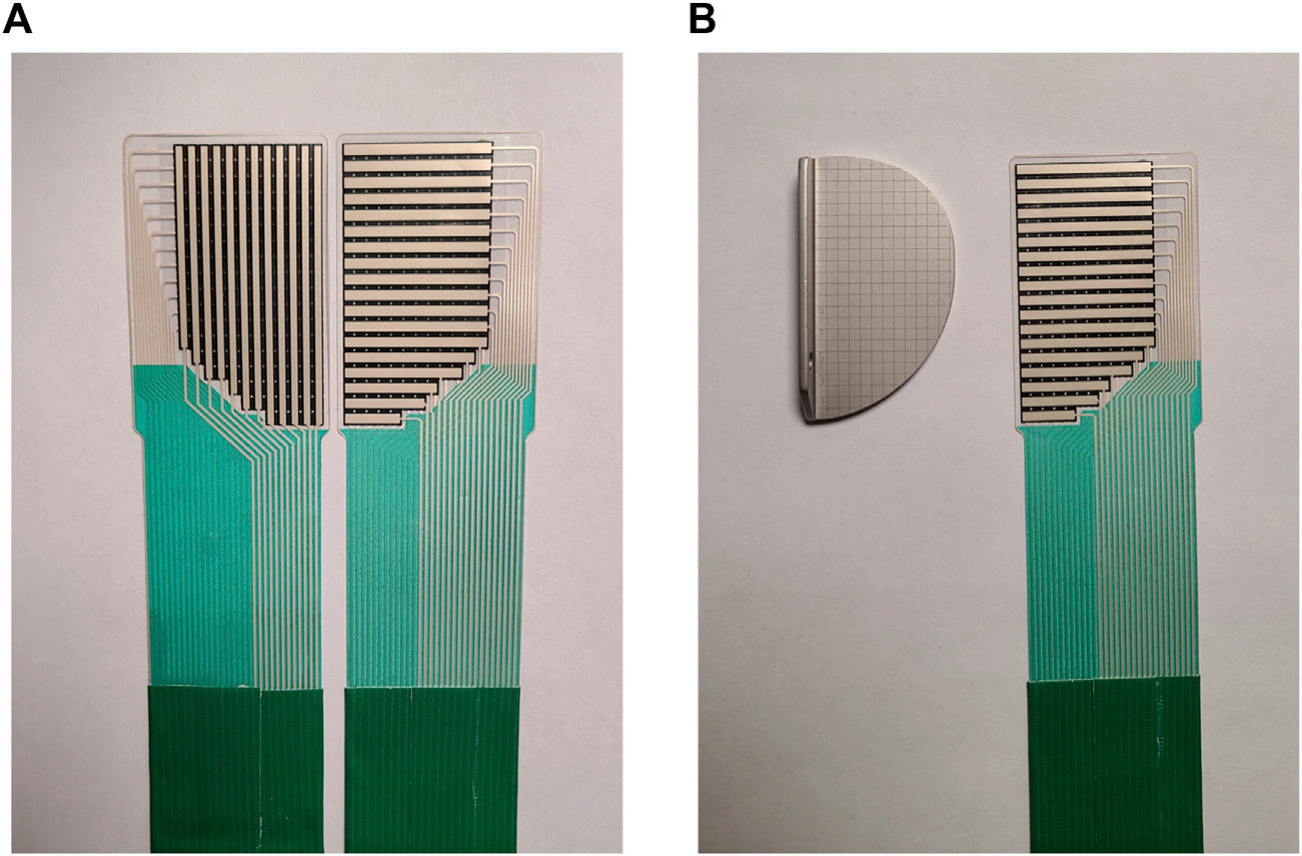
**(A)** Pressure sensors designed for the Oxford unicompartmental knee arthroplasty (anterior view and posterior view). The superior is a matrix sensing area with 197 measurement points and the inferior outputs pressure data via a transducer connected to a PC. **(B)** The pressure measuring area of the sensor corresponds to the shape of the tibial prosthesis (on the left is the trial mould of the tibial prosthesis, on the right is the customized sensor).

**FIGURE 2:**
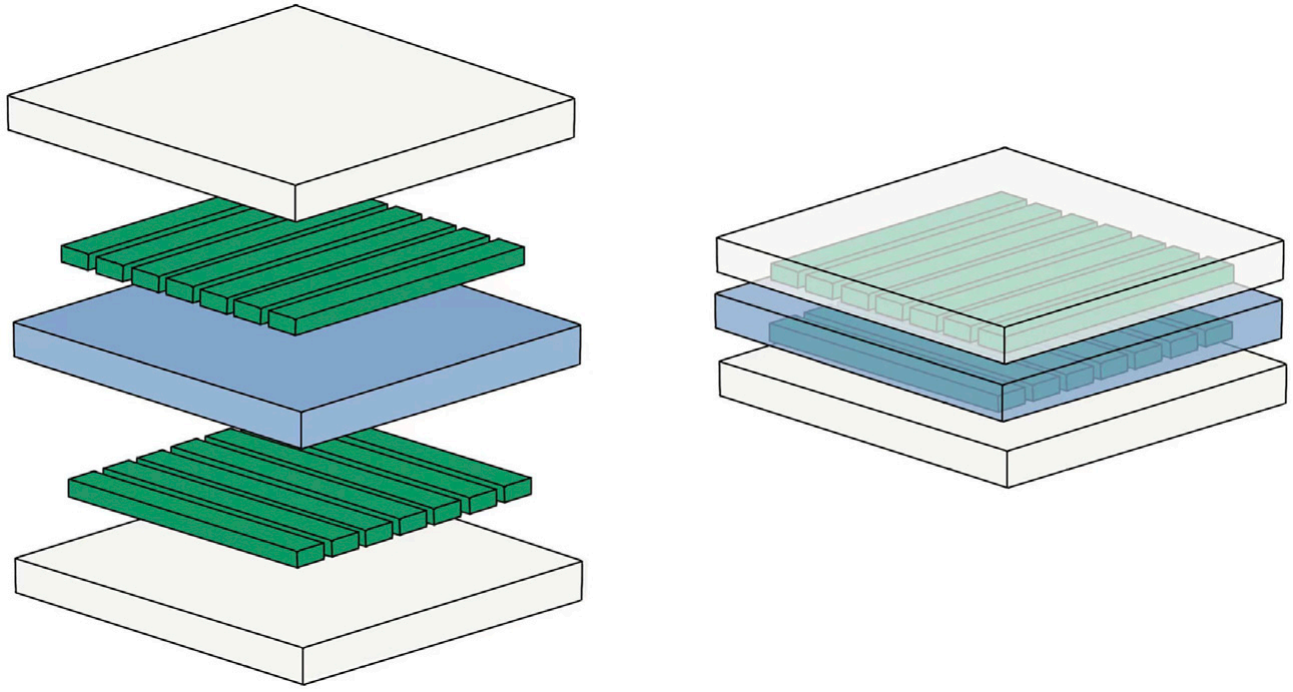
Model diagram of the sensor structure: the white transparent layer refers to the insulating layer; the central blue layer refers to the pressure-sensitive material; the green columns on both sides refer to the electrodes.

According to the characteristics of the OUKA prosthesis, a force sensor has been customised for inter-prosthetic pressure measurement. The pressure sensing area of this sensor is consistent with the shape of the tibial prosthesis, with a thickness of 0.12 mm, a sensing area of 45 × 22 mm^2^, and a total of 197 force-measuring points (Figure 1). The structure of the sensor is that the electrodes are sprayed in two pieces flexible materials vertically and horizontally, and the middle is lined with pressure-sensitive materials (Figure 2). A matrix network is formed by crossing horizontally and vertically, which can convert different pressures on the network nodes into resistance values, and finally output specific pressure values through the transducer. The data acquisition frequency can be up to 20Hz and the measuring range is 500N/cm^2^. We evaluated the accuracy and repeatability of this sensing technology through cadaver specimen measurements in the early stage and found that the error between the calibration value and the measured value was within 1%, and the error/mean ratio of repeated measurements was also 1% Below: indicates good accuracy and repeatability (Sun et al., 2021; Sun et al., 2022). Teflon (Polytetrafluoroethylene) tape (0.1 mm in thickness) was used to fix it to reduce the influence of shear force, and a STERRAD low-temperature sterilizer (Johnson&Johnson, Inc, United States) was used for hydrogen peroxide plasma low-temperature disinfection, and data calibration was performed before and after disinfection. It was found that low-temperature disinfection below 60°C had no significant effect on data acquisition, which is consistent with another common resistive matrix sensor (Agins et al., 2003).

### 2.3 Surgery and intraoperative measurements

**FIGURE 3:**
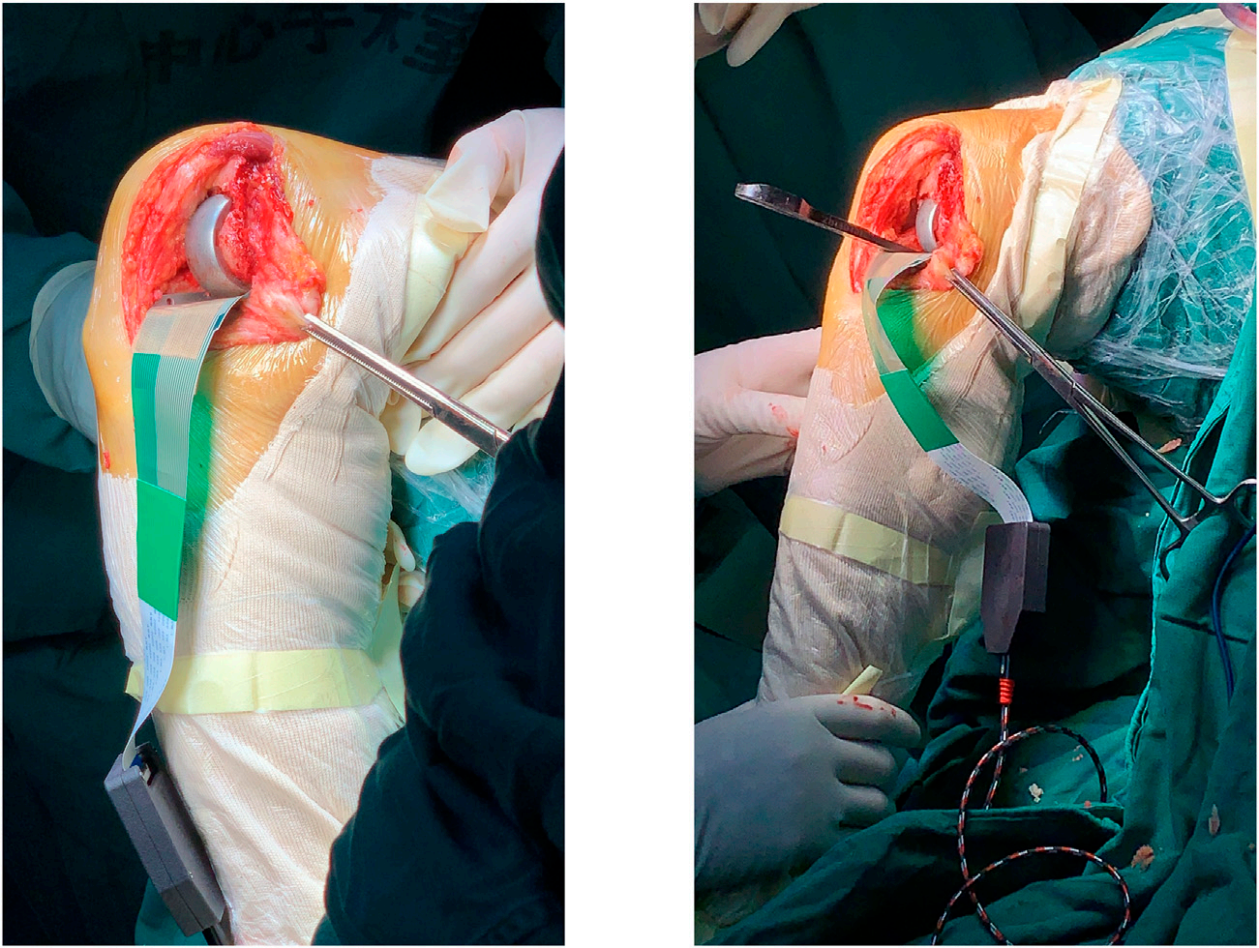
Intraoperative pressure measurement: the sensor is attached to the surface of the tibial prosthesis trial mould, while the femoral prosthesis trial mould and gap block are placed.

All operations on included patients were performed by the same group of surgeons, and the chief surgeon was a senior expert with a total of more than 2,000 UKA operations. The surgery is performed under general or spinal anesthesia. A medial parapatellar approach was selected. First, the tibial osteotomy was performed under extramedullary guidance, with the posterior tilt angle set at 7° in principle. If the pre-operative lateral x-ray shows that the original PTS is too large or too small, it should be adjusted to an appropriate situation. Femoral side preparation begins with the identification of the component positioning holes, followed by the posterior condylar osteotomy. A mould was fitted to test the differences in flexion and extension gaps. Grinding the distal femur until the 20° extension gap was confirmed to be in balance with the 90° flexion gap. Then, a slot is made in the tibial osteotomy surface for the tibial prosthesis keel. Finally, a trial mould was fitted to confirm proper ligament tension and knee stability. At the same time, check that the knee can be fully extended to 0° without hyperextending. The sensor was fixed on the surface of the tibial prosthesis trial mold with Teflon tape (0.12 mm thickness), and a space block of appropriate size was placed. The sensor was connected to the sensor and PC, and the pressure between the prosthesis test mold and the space block was recorded at the knee joints of 120°, 90°, 60°, 45°, 20°, and 0° respectively (Figure 3). The data is acquired by self-developed multi-array pressure sensor acquisition software and recorded in a txt file for statistical analysis. The data acquisition frequency was set at 10 Hz for 5 s. For each angle a total of 50 data were obtained and the mean value was taken as the inter-prosthetic pressure.

### 2.4 Clinical and radiographic evaluation

Clinical evaluation: All patients were followed up for at least 6 months. The following indicators were collected before surgery and at the last follow-up: knee joint range of motion (ROM), residual fixed flexion deformity (fixed flexion degree, FFD) when the knee joint was fully extended (if there was no residual flexion deformity, is recorded as 0°), and Knee Society Score (KSS).

**FIGURE 4:**
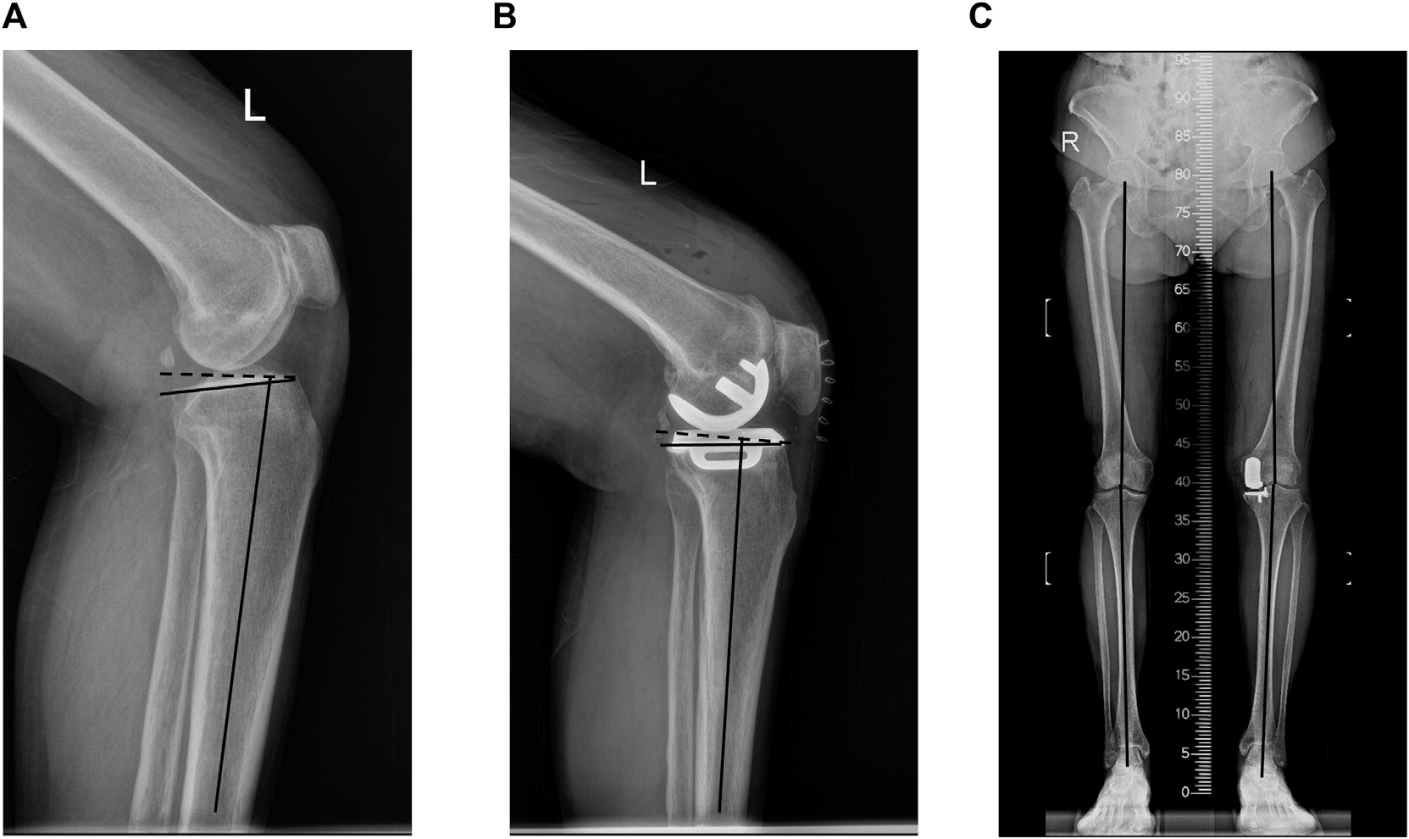
**(A)** Preoperative posterior tibial slope. **(B)** Postoperative posterior tibial slope. **(C)** hip–knee–ankle angle (HKAA).

Radiographic evaluation: Standard frontal, lateral, and full-length weight-bearing X-ray films of the knee joint were taken before the operation and within 1 week after the operation. The measurement indicators include: preoperative posterior tibial slope ( ${PTS}_{pre}$ ) defined as the angle between the vertical line of the tibial axis on the lateral view of the knee joint and the tibial plateau; postoperative posterior tibial slope ( ${PTS}_{post}$ ), defined as the angle between the vertical line of the tibial axis on the postoperative lateral film and the tangent line of the tibial prosthesis; hip-knee-ankle angle (HKAA), defined as the center of the hip-knee joint on the full-length film of the lower limb he angle between the line and the knee-ankle center line (Figure 4).

### 2.5 Calculation method

Measure the inter-prosthesis pressure of the knee joint at 0°, 20°, 45°, 60°, 90°, and 120° of knee flexion, and record them as $F_{0{}^{\circ}}$ , $F_{20{}^{\circ}}$ , $F_{45{}^{\circ}}$ , $F_{60{}^{\circ}}$ , $F_{90{}^{\circ}}$ , $F_{120{}^{\circ}}$ . Continuous variable are presented as mean ± standard deviation. Since the Oxford unicompartmental knee arthroplasty requires the balance of the inter-prosthesis pressure between 20° and 90° of knee flexion, we calculated the average value of the inter-prosthesis pressure of 20°, 45°, 60°, and 90° of knee flexion as the mean inter-prosthesis pressure ( $F_{mean}$ ). In order to reduce the influence of inter-prosthesis pressure differences on the results, the inter-prosthesis pressure at the end of knee extension is expressed as the ratio of the inter-prosthesis pressure at 0° of knee extension (the knee joint is fully extended) to the average inter-prosthesis pressure, and the formula is:

$$F_{extension}=\frac{F_{0{}^{\circ}}}{F_{mean}}\times 100\%$$

Similarly, the formula for the inter-prosthesis pressure at the end of the knee flexion is:

$$F_{flexion}=\frac{F_{120{}^{\circ}}}{F_{mean}}\times 100\%$$

The gap balance difference is defined as $F_{balance}$ , and the calculation formula is:

$$F_{balance}=F_{20{}^{\circ}}-F_{90{}^{\circ}}$$

The calculation formula for the change of tibial retroversion before and after the operation is:

$$∆PTS={PTS}_{post}-{PTS}_{pre}$$

Negative values indicate that postoperative PTS decreased compared with preoperative, while positive values indicated that postoperative PTS increased compared with preoperative.

### 2.6 Statistical analysis

Statistical analysis was performed using SPSS statistics25 (IBM Corp, Armonk, NY). The continuous variables were presented as means and standard deviations (SD), while the categorical variables were given as frequencies. The Pearson correlation coefficient test was used to evaluate the relationship between the inter-prosthesis pressure at various angles of the knee joint during the operation and the postoperative HKAA. At the same time, the correlation between $F_{extension}$ , $F_{flexion}$ , and ∆PTS, the correlation between ∆PTS and postoperative ROM, FFD, and the correlation between $F_{extension}$ and ROM were also evaluated by Pearson correlation coefficient test. Preoperative and postoperative imaging indicators (HKAA, PTS) and clinical evaluation indicators (ROM, FFD, KSS) were compared by independent sample *t*-test, and *p* < 0.05 was considered statistically significant.

## 3 Results

### 3.1 Variation trend of inter-prosthesis pressure in different knee joint angles and gap balance difference distribution

**FIGURE 5:**
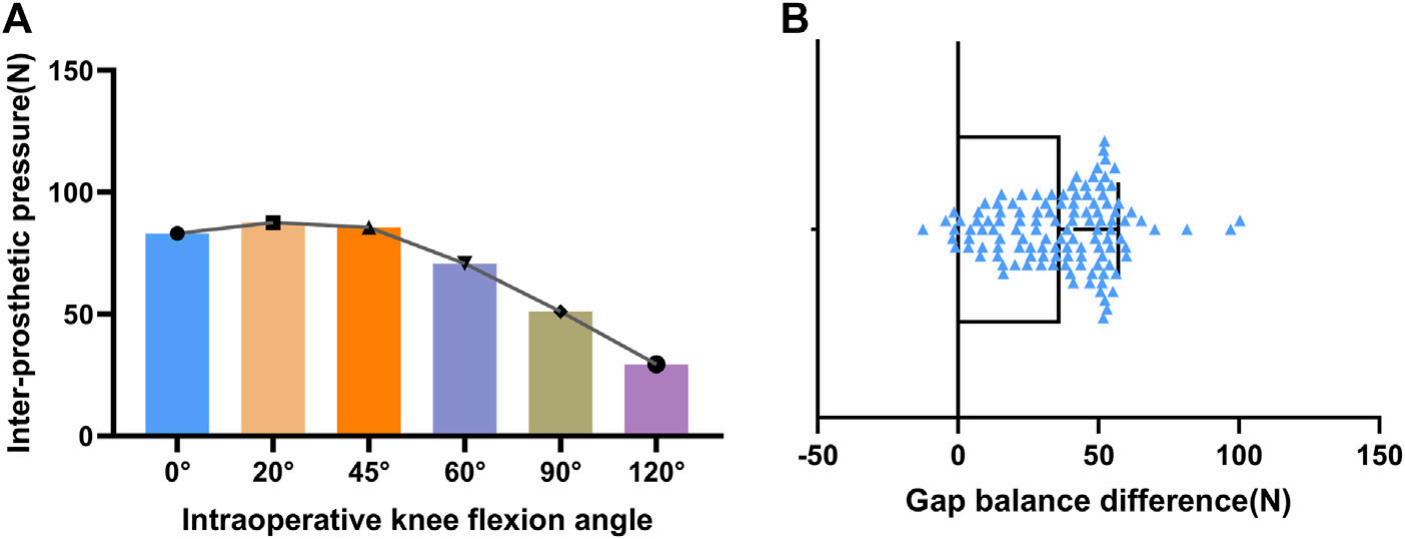
**(A)** variation trend of inter-prosthesis pressure in different knee joint angles. **(B)** gap balance difference distribution.

The inter-prosthesis pressures in the knee joint space during operation were: $F_{0{}^{\circ}}$ : 83.03 ± 49.94N, $F_{20{}^{\circ}}$ : 87.51 ± 44.43N, $F_{45{}^{\circ}}$ : 85.54 ± 50.86N, $F_{60{}^{\circ}}$ : 70.66 ± 47.15N, $F_{90{}^{\circ}}$ : 51.03 ± 33.67N, $F_{120{}^{\circ}}$ : 29.48 ± 26.40N. After calculation, the average inter-prosthesis pressure is: 73.68.28 ± 41.65N; after adjusting the average pressure, the relative pressure of $F_{extension}$ is 115.02% ± 64.72%, and the relative pressure of $F_{flexion}$ is 42.64% ± 27.96%. $F_{balance}$ is 36.48 ± 20.58N (Figure 5).

### 3.2 Correlation between inter-prosthesis pressure and lower limb alignment

**FIGURE 6:**
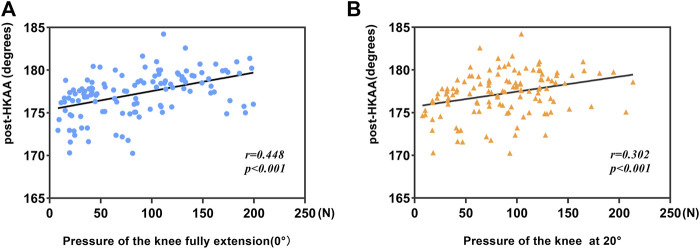
**(A)** Correlation between postoperative HKAA and pressure at 0°. **(B)** Correlation between postoperative HKAA and pressure at 20°.

Inter-prosthesis pressure at 0° was positively correlated with postoperative HKAA in the Pearson correlation coefficient test (*r* = 0.448, *p* < 0.001). There was also a positive correlation between the inter-prosthesis pressure at 20° and postoperative HKA (*r* = 0.302, *p* < 0.001), while the inter-prosthesis pressure at other angles did not correlate with postoperative HKAA (Figure 6; Table 2).

**TABLE 2:** Correlation between inter-prosthetic pressure at different angles and postoperative HKAA.

Variables	Post-HKAA (postoperative hip–knee–ankle angle)	
	*r*	*p-value*
$F_{0{}^{\circ}}$	0.448	< 0.001
$F_{20{}^{\circ}}$	0.302	< 0.001
$F_{45{}^{\circ}}$	0.092	0.316
$F_{60{}^{\circ}}$	0.110	0.230
$F_{90{}^{\circ}}$	0.132	0.148
$F_{120{}^{\circ}}$	0.036	0.696

**FIGURE 7:**
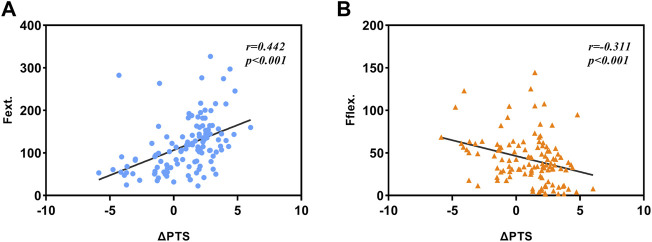
**(A)** Correlation between ΔPTS and $F_{extension}$ . **(B)** Correlation between ΔPTS and $F_{flexion}$ .

In terms of sagittal alignment, ΔPTS was positively correlated with $F_{extension}$ (*r* = 0.442, *p* < 0.001), and negatively correlated with $F_{flexion}$ (*r* = −0.311, *p* < 0.001) (Figure 7). Further analysis found that ΔPTS have no correlation with postoperative ROM (*r* = 0.047, *p* > 0.05).

### 3.3 Radiological and clinical outcomes

The UKA operation of the included patients was carried out smoothly, and no infection, bearing dislocation, or other complications occurred. Postoperative HKAA was significantly increased compared with preoperative (*p* < 0.05), PTS was not significantly different from preoperative, ROM was significantly improved compared with preoperative (*p* < 0.001), and FFD was significantly improved compared with preoperative (*p* = 0.001). Postoperative KSS clinical score and functional score were significantly improved compared with preoperative (*p* < 0.001) (Table 3).

**TABLE 3:** Comparison of radiological and clinical outcomes.

	Preoperative	Postoperative	*p*-value
HKAA	172.32 ± 3.40	177.25 ± 2.58	<0.001
PTS	8.27 ± 3.08	8.59 ± 1.84	0.329
ROM	117.98 ± 9.08	123.97 ± 6.48	<0.001
FFD	3.18 ± 4.15	0.66 ± 1.81	<0.001
KSS clinical score	48.25 ± 7.73	88.34 ± 4.24	<0.001
functional score	47.09 ± 2.93	80.33 ± 4.44	<0.001

HKAA = hip-knee-ankle angle; PTS = posterior tibial slope; ROM = range of motion; FFD = fixed flexion deformity; KSS = knee society score.

## 4 Discussion

This study is the first to use sensor technology to describe the change of inter-prosthesis pressure in different knee angles in OUKA. At the same time, the difference in inter-prosthesis pressure was used to quantify the gap balance. Through the analysis of inter-prosthesis pressure and postoperative lower limb alignment index, we found that the inter-prosthesis pressure at 0° and 20° is related to postoperative HKAA, which can reflect postoperative HKAA to some degree. The change of PTS will lead to the change of the inter-prosthesis pressure between the end of knee extension and knee flexion. The excessive increase of PTS will not increase the range of motion of the knee joint after surgery but may lead to limitation of knee extension.

In this study, we measured the OUKA inter-prosthesis pressure achieved by a surgeon who performed more than 2,000 OUKA operations and operated in strict accordance with the standard surgical procedures and initially formed a multi-angle inter-prosthesis pressure reference. The inter-prosthesis pressure is minimal at deep flexion of the knee joint, increases gradually as the joint is extended, reaches a peak at 20°–45°, and further decreases at full extension. This may be due to the single-radius design of the Oxford UKA prosthesis, while the radius and center of the human femoral condyle flexion-extension articular surface are not the same (Lu et al., 2021). After installation of the prosthesis, the spherical surface of the single-radius femoral prosthesis will partially exceed the original articular surface when the knee is flexed to a certain degree (probably 20°–45°), so there may induce an increase in the inter-prosthesis pressure in the middle of flexion. While the pressure at 0° is higher than 60°, it may be caused by the soft tissue around the knee joint being stretched after the knee joint is fully extended (Zhou et al., 2018).

Postoperative valgus malalignment with overcorrection (HKAA>180°) after UKA is the most common cause of increased lateral compartment load and leading to osteoarthritis progression (Murray et al., 1998; Pandit et al., 2016; Zhang et al., 2019). However, in addition to the use of navigation or robotic surgery (Innocenti and Bori, 2021; Kort et al., 2022), in traditional surgery, judging whether the force line is everted still relies solely on the surgeon’s visual inspection. According to our research results, there is a positive correlation between the inter-prosthesis pressure of the knee joint at 0° and 20° and postoperative HKAA, while the pressure at other angles has no correlation. The straightened inter-prosthesis pressure directly measures the inter-prosthesis pressure in the straightened state, which can reflect the degree of correction of the force line of the lower limbs to a certain extent. However, many factors may affect the gap pressure during knee extension (Innocenti et al., 2014). For example, in the knee joint with flexion deformity before the operation, the tension of the posterior joint capsule is often high, so that the gap contact force is generally higher when the knee is extended (Malavolta and Kley, 2021). Except at 0° and 20°, the position of the knee at other angles is close to or in flexion. At this point, the tibial plateau is in contact with the posterior femoral condyle, rather than the distal femoral articular surface. The soft tissues surrounding the knee joint are also in a relatively relaxed state. Inter-prosthetic pressures in this state are therefore difficult to predict the hip-knee-ankle angle (HKAA), which is measured during knee extension.

Gap balancing is an important surgical technique step in the OUKA procedure. The purpose is to keep the soft tissue around the knee joint in proper tension during the flexion and extension process of the knee joint, so as to avoid instability during the postoperative knee joint movement. The ideal balance of the flexion-extension gap should be the same inter-prosthesis pressure in flexion-extension gap, but this process will inevitably be affected by many factors, such as the patient’s position. At the same time, we found that the gap balance difference within a certain range will not affect the early clinical effect of patients after surgery (Ge et al., 2023).

PTS changes affect the biomechanics of the lower limb and the clinical outcome after UKA. The Oxford unicompartmental prosthesis did not consider the anatomical difference of the knee joint, and the PTS was uniformly set at 7°. But in fact, the distribution of PTS individuation is very wide. By observing the CT examination results of 2031 patients with medial UKA, Nunley found that the preoperative PTS distribution ranged from −9.6° to 16.8°, which has a very wide distribution (Nunley et al., 2014), and the standard 7° retroversion may have a great change in some patients. Excessive PTS angles can gradually increase the stress of the posteromedial tibial cortex and cancellous bone, increasing the risk of prosthetic loosening, posterior tibial collapse, and even anterior cruciate ligament rupture (Aleto et al., 2008; Gulati et al., 2009; Suero et al., 2012). Most of the previous studies focused on the impact of postoperative PTS on the clinical effect of UKA, but the conclusions were not consistent. Clarius et al. found that 88% of postoperative PTS after UKA were within the recommended range, but whether it exceeded the recommended range did not seem to affect the short-term clinical outcomes of patients (Clarius et al., 2010). However, Hernigou et al. found that postoperative PTS exceeding 7° would increase the UKA revision rate (Hernigou and Deschamps, 2004). Aleto et al. found that in patients with posterior tibial plateau collapse, the average postoperative PTS was 12° ± 2°, which was significantly greater than that in the control group (Aleto et al., 2008). In terms of knee joint mobility, Inui et al. found that postoperative PTS increase was associated with high postoperative knee flexion, and the postoperative clinical effect of the high-flexion knee joint was better, especially the patient-reported effect (Inui et al., 2020). These inconsistent results may be due to investigators’ excessive focus on postoperative PTS. The PTS in its natural state should be considered during the operation to avoid excessive changes that may lead to poor postoperative clinical results. This study, it found that changes in the PTS affect the extension and flexion gaps. Excessively increasing the PTS may cause the extension gap to be too tight, which is not conducive to the knee extension exercise of postoperative patients. However, the reduction of PTS will lead to a too-tight flexion gap, which is not conducive to the patient’s deep knee flexion. At the same time, increasing the PTS did not increase the knee range of motion in patients.

Our study also has some limitations. This study is based on the collection of surgical data from the same orthopedic surgeon, and the establishment of standards for gap pressure and gap balance requires large samples and multi-center data collection. The collection of pressure data is carried out on the trial model, and the final tibial side prosthesis needs to be fixed with bone cement. The thickness of bone cement may affect the pressure value between the actual prosthesis, but in this study, Teflon tape and the sensor itself have a certain thickness, which can offset the effect of the bone cement to some extent.

## 5 Conclusion

In this study, a customized sensor was used to measure the inter-prosthesis pressure during OUKA surgery, and the results showed that the inter-prosthesis pressure was not consistent at different angles. At the same time, we further explored the relationship between pressure and lower limb alignment after OUKA and found that the inter-prosthesis pressure measured in the extended position can predict postoperative HKAA to a certain extent. Changes in PTS will affect the inter-prosthesis pressure at the end of knee flexion and knee extension, but this change is not related to the range of motion of the knee joint.

## Data Availability

The original contributions presented in the study are included in the article/Supplementary Material, further inquiries can be directed to the corresponding authors.
